# Phytochemical Constituents, Health Benefits, and Industrial Applications of Grape Seeds: A Mini-Review

**DOI:** 10.3390/antiox6030071

**Published:** 2017-09-15

**Authors:** Zheng Feei Ma, Hongxia Zhang

**Affiliations:** 1 Department of Public Health, Xi’an Jiaotong-Liverpool University, Suzhou 215123, China; 2 Department of Food Science, University of Otago, Dunedin 9054, New Zealand

**Keywords:** grape seeds, grapes, proanthocyanidins, flavonoids, catechins

## Abstract

Grapes are one of the most widely grown fruits and have been used for winemaking since the ancient Greek and Roman civilizations. Grape seeds are rich in proanthocyanidins which have been shown to possess potent free radical scavenging activity. Grape seeds are a complex matrix containing 40% fiber, 16% oil, 11% proteins, and 7% complex phenols such as tannins. Grape seeds are rich sources of flavonoids and contain monomers, dimers, trimers, oligomers, and polymers. The monomeric compounds includes (+)-catechins, (−)-epicatechin, and (−)-epicatechin-3-O-gallate. Studies have reported that grape seeds exhibit a broad spectrum of pharmacological properties against oxidative stress. Their potential health benefits include protection against oxidative damage, and anti-diabetic, anti-cholesterol, and anti-platelet functions. Recognition of such health benefits of proanthocyanidins has led to the use of grape seeds as a dietary supplement by the consumers. This paper summarizes the studies of the phytochemical compounds, pharmacological properties, and industrial applications of grape seeds.

## 1. Introduction

Grapes are one of the most widely grown fruits and the total production of grapes worldwide is approximately 60 million tons [1]. The major producers of grapes are the USA, China, Italy, and France [1]. Grapes can be categorized into grapes with edible seeds, seedless, wine grapes, table grapes, and raisin grapes [2]. North American grapes (*Vitis labrusca* and *Vitis rotundifolia*), European grapes (*Vitis vinifera*), and French hybrids are the main species of grapes [2].

Grapes have been used for winemaking since the ancient Greek and Roman civilizations [3]. In the winemaking process, over 0.3 kg of solid byproducts are produced per kg of grapes crushed [4]. Grape marc is the main byproduct which accounts for about two third of the solids [4]. Grape marc consists of grape skins (50%), seeds (25%), and stalks (25%) [4]. Therefore, grape seeds are the industrial byproduct of the winemaking process. Although grape seeds are a relatively inexpensive source of antioxidant compounds [5], they amount to 38–52% on a dry matter basis [6]. Grape seeds are treated as waste if extracts are not made and it is estimated that about 10–12 kg of grape seeds in 100 kg of wet residues are produced by the industry [1].

In recent years, grape seed extract has become increasingly popular on the market as a nutritional supplement especially in the Australia, Korea, Japan and the United States [7]. This is because grape seeds are rich in phenolic compounds [8] and have potentially beneficial effects for human health such as protection against peptic ulcers [8,9]. Grape seeds have been reported to exhibit scavenge superoxide radicals [10]. Grape seeds are rich in flavan-3-ol, including proanthocyanidins and catechins [8]. They contain high concentration of polyphenol proanthocyanidins, which are the oligomers of flavan-3-ol units including catechin and epicatechin [11]. Proanthocyanidins, which belong to condensed tannins, are present as procyanidins and prodelphinidins in grape seeds and skins [12]. Procyanidins and prodelphinidins are extracted from the seeds and skins during fermentation into wine [12]. Anthocyanins and proanthocyanidins play a significant role in the stability, taste, and color of the red wines [13]. The simplest proanthocyanidins are dimeric proanthocyanidins which have 4→8 linked monomers [14].

Therefore, the aim of this work was to summarize the phytochemical constituents, pharmacological activities, and industrial applications of grape seeds. We hope that this work would be a valuable reference resource to provide beneficial inspiration for the future studies.

### Search Strategy

An electronic literature search was conducted using PubMed, Medline (OvidSP), and Google Scholar until May 2017. Additional articles were identified from references in the retrieved articles. Search terms included combinations of the following: “grape”, “grape seed”, “phytochemical”, “antioxidant”, “anthocyanins”, “proanthocyanidins”, and “pharmacological”. The search was restricted to articles in English that addressed the phytochemical constituents and pharmacological properties of grape seeds. For the purpose of this review, studies involving human subjects will be given priority.

## 2. Phytochemical Compounds in Grape Seeds

Grape seeds contain protein (11%), fiber (35%), minerals (3%), and water (7%) [15]. In addition, the lipid content of grape seeds ranges from 7 to 20% [15]. The oil content of grape seeds is traditionally extraction using mechanical methods or organic solvents [4]. In mechanical extraction, although the quality of product is superior, the extraction gives a lower yield. While organic solvent extraction gives a higher yield, it requires solvent recovery through distillation and the final product contains traces of residual solvent [4]. While the supercritical method is regarded as a promising method which can produce similar quantity and better quality of oil yield than mechanical and organic solvent extractions [4]. Cold-pressing is used to extract the oil from grape seeds without chemical treatment or heat [16]. Although the cold pressing usually gives a lower yield than other conventional solvent extraction, it may retain more bioactive components and be safer because there are no solvent residues in the grape seed oil [15].

Several studies have been conducted on grape seeds, in order to determine their bioactive compounds [8,17,18,19,20,21]. Grape seed extracts contain a heterogeneous mixture of monomers (5–30%), oligomers (17–63%), and polymers (11–39%) composed of proanthocyanidins [22]. Proanthocyanidins are the major compound in grape seed extracts [23]. The red color and astringent taste of grape seed extracts can be attributed to proanthocyanidins. However, higher concentrations of proanthocyanidins may affect the sensory and color properties of the product [24].

Negro et al. [25] reported that the total phenols (gallic acid equivalent (GAE)), total flavonoids (catechin equivalent (CE)), catechin equivalent (CE), and proanthocyanidins (cyanidin equivalent (CyE)) in red grape seed extracts were 8.58 g/100 g dry matter (DM), 8.36 g/100 g DM, 6.41 g/100 g, DM and 5.95 g/100 g DM, respectively. Another study by Ghouila et al. [26] documented that the total phenolic content and total flavonoid content of Ahmeur Bouamer grape seeds were 265.15 mg GAE/g of dry mass and 14.08 mg catechin equivalent (CE)/g of dry mass, respectively. 

In a study analyzing 26 samples of six white grape varieties and 44 of four red grape varieties from different areas of Castilla-La Mancha, Spain, Rodríguez Montealegre et al. [8] reported that the grape seeds were shown to contain catechin, epicatechin, epicatechin gallate, protocatechic acid, procyanidin B1, procyanidin B2, procyanidin B3, and procyanidin B4. Small quantities of galic acid and protocatechic acid were also present in grape seeds [8].

Similarly, another study by Escribano-Bailon et al. [17] reported that the major constituents in *V. vinifera* (Tintal del pais) grape seeds were (+)-catechin (11%) followed by (−)-epicatechin (10%), (−)-epicatechin-3-*O*-gallate (9%), epicatechin 3-O-gallate-(4β→8)-catechin (B1-3-O-gallate) (7%), and epicatechin-(4β→8)-epicatechin (dimer B2) (6%). These results were also similar with previous studies conducted on the polyphenols of grape seeds from other varieties of grape species in Greek islands [21,27,28]. Anastasiadi et al. [21] reported that the total phenolic content in grape seeds ranged from 325 to 812 mg/g gallic acid equivalents.

There are 14 dimeric, 11 trimeric procyanidins, and 1 tetrameric procyanidin that have been identified from grape seeds [18]. In a study that investigated the quantities of flavan-3-ols in the seeds of 17 grape cultivars grown in Ontario, Canada using high-performance liquid chromatography (HPLC), Fuleki and Ricardo da Silva [18] documented the presence of monomers of (+)-catechin, (−)-epicatechin-3-O-gallate, and (−)-epicatechin. Considerable quantities of highly polymerized procyanidins were also found in grape seeds [18]. Similarly, Prieur et al. [29] also reported the presence of catechin, epicatechin-3-O-gallate, and epicatechin were determined by HPLC following the thiolysis degradation, providing evidence that procyanidins are the grape seed tannins.

Gabetta et al. [19] documented the presence of monomers, dimers, trimers, tetramers, pentamers, hexamers, heptamer, and their gallates in grape seeds. A study by Freitas et al. [30] obtained several fractions of oligomeric and polymeric procyanidins from grape seeds using gel chromatography. The authors [30] also identified several grape seed flavan-3-ol derivatives and phenol acids including protocatechic, caffeic acids, trimer epi-(4β-8 or 6)-epi-(4β-6 or 8)-epi, trimer cat-(4α-8)-epi-(4β-8)-epi, and trimer cat-(4α-8)-epi-(4β-6)-cat.

Some factors such as the variety of grapevine, viticultural, and environmental factors can influence the concentration of phenolic compounds in grapes [8]. Epicatechin, (+)-catechin, and gallic acid were the major phenolic compounds detected in the muscadine grape seeds [20]. Pastrana-Bonilla et al. [20] reported that the concentration of epicatechin, (+)-catechin, gallic acid, total anthocyanin, and total phenolics in muscadine grape seeds were 1299 mg/100 g of fresh weight (FW), 558 mg/100 g of FW, 6.9 mg/100 g of FW, 4.3 mg/100 g of FW, and 2179 mg/g GAE, respectively.

## 3. Pharmacological Properties

Proanthocyanidins are present in substantial amounts in grape seeds and have attracted attention of consumers because of their potential health effects [31]. In vitro, proanthocyanidins have been shown to exhibit strong antioxidant activity and scavenge reactive oxygen and nitrogen species, modulate immune function and platelet activation, and produce vasorelaxation by inducing nitric oxide (NO) release from endothelium [31]. In addition, proanthocyanidins also inhibits the progression of atherosclerosis and prevent the increase of low-density lipoprotein (LDL) cholesterol concentration [32].

### 3.1. Anti-Diabetic

Montagut et al. [33] reported Wister female rats treated with 25 mg grape seed procyanidin extract/kg body weight per day for 30 days had an improved homeostatis model assessment-insulin resistance index accompanied by downregulation of primers Glut4, Irs1, and Pparg2 in mesenteric white adipose tissue (WAT), suggesting that grape seed procyanidin has a positive long term-effect on glucose homeostasis.

Another study by Montagut et al. [34] demonstrated that the oligomeric structures of grape seed procyanidin extracts activated the insulin receptor by interacting and inducing the autophosphorylation of the insulin receptor in order to stimulate the glucose uptake.

### 3.2. Antioxidant

Different methods including the 1,1-diphenyl-2-picryhidrazyl (DPPH) method and oxygen radical absorbance capacity (ORAC) have been employed to evaluate the antioxidant capacity of phenolic compounds from grape seeds. Poudel et al. [35] reported that the antioxidant capacity of grape seed extract using DPPH ranged between 17 and 92 mmol Trolox^®^ antioxidant equivalent (TE)/g. While the ORAC method was 42 mmol TE/g [35]. Pastrana-Bonilla et al. [20] demonstrated that the antioxidant capacity was the highest in grape seeds (281 μM Trolox equivalent antioxidant capacity (TEAC)/g of FW) followed by leaves (236 μM TEAC/g of FW), skins (13 μM TEAC/g of FW), and pulps (2.4 μM TEAC/g of FW), suggesting that the grape seeds extracts are promising antioxidant for dietary supplement. Similarly, two studies [36,37] also confirmed the antioxidant activity of grape seed extract by using β-carotene linoleate, linoleic acid peroxidation, DPPH, and phosphomolybdenum complex methods.

Puiggròs et al. [38] reported that grape seed procyanidin extracts modulated the expression of antioxidant systems, suggesting that grape seed procyanidin extracts might improve the cellular redox status via glutathione synthesis pathways. Another study by Guo et al. [39] reported that grape seed procyanidins provided protective effect against the ethanol induced toxicity in mouse brain cells. Vinson et al. [40] reported that the supplementation of 50 mg/kg and 100 mg/kg grape seed proanthocyanidin extracts in hamsters reduced the formation of foam cells by 50% and 63%, respectively. The development of foam cells is the indicator of early stages of atherosclerosis [40]. In a randomized, double-blind, placebo-controlled study, Preuss et al. [41] reported that the supplementation of grape seed proanthocyanidin extracts and chromium polynicotinate in hypercholesterolemic participants decreased the concentrations of total cholesterol, oxidized LDL, and LDL significantly after two months.

### 3.3. Anti-Platelet

Grape seed extracts were shown to exhibit anti-platelet action [42]. Olas et al. [42] reported that grape seed extracts (5–50 μg/mL) showed a reduction in platelet aggregation, adhesion, and generation of superoxide anion. In addition, the authors [42] also found that grape seed extract was more effective than the pure resveratrol solution in the reduction of platelet processes.

### 3.4. Anti-Cholesterol

Cetin et al. [43] reported that there were no significant changes in levels of superoxide dismutase and catalase of rats supplemented with grape seed extracts when methotrexate (MTX) was administered. The authors [43] suggested that the supplementation of grape seed extract may be protective against oxidative damage induced by MTX which is used as cytotoxic chemotherapeutic agent [44].

In a 12-week of supplementation containing 0, 200, or 400 mg grape seed extract to 61 healthy subjects with LDL cholesterol level of 100 to 180 mg/dL, Sano et al. [45] reported that the 200 mg and 400 mg group reported a significant decrease in malondialdehyde-modified LDL (MDA-LDL) when compared to the baseline level after 12 weeks (*p* = 0.008 and 0.009, respectively). MDA-LDL is involved in the pathogenesis of arteriosclerosis [46].

In a study that investigated the effect of supplementing a single high-fat meal with 300 mg of proanthocyanidin-rich grape seed extracts in eight male adults, Natella et al. [47] reported that grape seed extracts reduce postprandial oxidative stress by increasing the plasma antioxidant concentration and preventing the increase of lipid hydroperoxides. Consequently, this improves the resistance to oxidative modification of LDL cholesterol [47]. It is suggested that the polyphenols in grape seed extracts activate serum paraoxonase (PON) which prevents the postprandial increase in lipid peroxides [47]. Serum paraoxonase is an enzyme associated with high-density lipoprotein (HDL) that hydrolyzes lipoprotein peroxides and inhibits LDL oxidation [48].

### 3.5. Anti-Inflammation

In a study that evaluated the effect of procyanidin from grape seeds on the inflammatory mediators in rat fed with a high fat diet, Terra et al. [49] reported that rats fed with a high fat diet supplemented with procyanidins from grape seeds (345 mg/kg feed) for 19 weeks had a lower plasma C-reactive protein (CRP) level than rat fed with high-fat diet, suggesting that the decrease in plasma CRP is related to a downregulation of CRP mRNA expression in the liver and mesenteric white adipose tissue (WAT). In addition, the authors [49] also reported a decrease in the expression of the proinflammatory cytokine tumour necrosis factor alpha (TNF-α) and interleukin 6 (IL-6) in the mesenteric WAT of rats fed with a high fat diet supplemented with procyanidins from grape seeds. IL-6 is a stress-induced inflammatory cytokine, which is directly implicated in atherogenesis [49]. 

Studies have revealed that the concentrations of proinflammatory mediators are elevated in the conditions such as insulin resistant states of diabetes and obesity [50]. In a study that investigated whether procyanidins play a role in modulating inflammation, Chacon et al. [51] reported that macrophage-like (THP-1) cell lines and human adipocytes (SGBS) pre-treated with grape-seed procyanidin extracts had a reduction in IL-6 and monocyte chemoattracting protein (MCP-1) expression after inflammatory stimulus. In addition, the authors [51] also reported that the translocation of NF-κB to the nucleus was partially inhibited in macrophage-like (THP-1) cell lines and human adipocytes (SGBS) pre-treated with grape-seed procyanidin extracts.

Terra et al. [52] reported that rats fed with grape-seed procyanidin extracts had a reduced body weight and plasmatic systemic markers of TNF-α and CRP. In addition, grape-seed procyanidin extracts also increased adiponectin expression and decreased IL-6 [52]. Grape-seed procyanidin extracts are also linked to a reduced expression of epidermal growth factor module-containing mucin-like receptor 1 (EMR1) (specific marker of macrophage F4/80), suggesting a reduced macrophage infiltration of WAT [52]. Therefore, the regular consumption of food containing procyanidins might help to prevent low-grade inflammatory-related diseases in obesity characterized by macrophage accumulation in WAT and abnormal cytokine production [52].

In a study that investigated the effect of grape seed procyanidins on glucose metabolism in streptozotocin-induced diabetic rats, Pinent et al. [53] reported that grape seed procyanidins stimulated the glucose uptake in L6e9 myotubes and 3T3-L1 adipocytes in a dose-dependent manner. In addition, grape seed procyanidins also stimulated glucose transported-4 translocation to the plasma membrane [53]. The authors [53] suggested that procyanidins possesses insulin-like effects in insulin sensitive cells. Therefore, all these findings suggested that procyanidins in grape seeds inhibited mRNA levels and decreased the risk of diseases associated to obesity and high fat diets.

### 3.6. Anti-Aging

In a study investigating the effect of grape seed extracts on the aged-associated accumulation of oxidative DNA damage in male albino rats of Wister strain, Balu et al. [54] reported that grape seed extracts inhibited the accumulation of age-related oxidative DNA damage products such as 8-hydroxy-2′-deoxyguanosine (8-OHdG) and DNA protein cross-links in spinal cord and in various brain regions including the striatum, cerebral cortex, and hippocampus.

Another study by Balu et al. [55] reported that lipid peroxidation and antioxidant defenses in male albino rats supplemented with grape seed extract were normalized, suggesting that grape seed extracts could be used to improve the antioxidant status and decreased the incidence of free radical-induced lipid peroxidation in the central nervous system of aged rats.

### 3.7. Anti-Microbial

Since grape seeds are rich sources of polyphenols, grape seeds have also shown promise as novel microbial agents. Jayaprakasha et al. [37] reported that the defatted grape seed extracts were shown to exhibit anti-bacterial effect against *Bacillus cereus* (*B. cereus*), *Bacillus subtilis* (*B. subtilis*), *Staphylococcus aureus* (*S. aureus*), *Bacillus coagulans* (*B. coagulans*), *Escherichia coli* (*E. coli*), and *Pseudomonas aeruginosa* (*P. aeruginosa*). In addition, the authors demonstrated that these extracts completely inhibited Gram-positive bacteria and Gram-negative bacteria at 850–1000 ppm and 1250–1500 ppm, respectively. A study by Ghouila et al. [26] demonstrated that grape seed extracts exhibited antimicrobial activity to *Microccocus luteus* ATCC^®^9341, *S. aureus* ATCC^®^29213, *E. coli* ATCC^®^25992, *P. aeruginosa* ATCC^®^27853, *Aspergillus niger*, and *Fusarium oxysporum*, with a diameter of the inhibition growth zone ranged from 15–20 mm.

Baydar et al. [56] reported that grape seed extracts acted against *S. aureus* after 48 h and *Aeromonas hydrophila* after 1 h. A study by Anastasiadi et al. [21] reported that the minimum inhibition concentration of grape seed extract against *Listeria monocytogenes* (*L. monocytogenes*) was 0.26, suggesting that grape seeds could be used as an inexpensive source of natural antilisterial mixtures. Future research should be conducted on screening the grape seed extracts for natural anti-microbial compounds.

Another study by Brown et al. [57] reported that muscadine grape seed extracts were effective in inhibiting *H. pylori* in vitro. The authors [57] demonstrated that although muscadine grape seed extracts had the highest total phenolic content (646 mg GAE/g dry weight (dw)) than skin extracts (135 mg GAE/g dw), the skin extracts had a better efficacy than seed extracts against *H. pylori* [57]. Therefore, it is suggested that the anti-*H. pylori* activity might be influenced by the type and content of phenolics [57]. *H. pylori* infection is associated with several gastroduodenal diseases such as gastric cancer [58].

### 3.8. Anti-Tumour

Grape seed extracts have also exhibited anti-tumor properties. Promising results have been shown by several studies performed in human colorectal carcinoma [59], head and neck squamous cell carcinoma [60], and prostate cancer cells [61]. Therefore, it is suggested that the supplementation of grape seed extracts might be an effective anti-tumor agent in clinical settings.

### 3.9. Other Pharmacological Properties

A study by Feng et al. [62] reported that grape seed extracts reduced brain weight loss from 20% in vehicle pups to 3% in treated pups (*p* < 0.01). The loss of brain weight was used to measure the brain damage in pups with neontal hypoxic ischemic brain injury. In addition, grape seed extract also improved the histopathologic brain score in cortex, hippocampus, and thalamus. The authors [62] suggested that the treatment with grape seed extracts could be used to inhibit lipid peroxidation and reduce hypoxic ischemic brain injury.

### 3.10. Toxicity

Grape seed extract has been approved as generally recognized as safe (GRAS) by Food and Drug Administration (FDA) and is sold commercially as a dietary supplement listed on the Everything Added to Food in the United States (EAFUS) database [63]. Bentivegna and Whitney [64] reported that no-observed-adverse effect level (NOAEL) of grape seed extracts determined in the rats was 1.78 g/kg body weight/day and the normal amount of grape seed extract used in food applications was 0.01–1%.

Sano et al. [45] also reported that there were no abnormal changes in the physiological and clinical laboratory tests in participants after taking tablets containing 200 mg and 400 mg grape seed extracts. In addition, no problematic results were reported in the urinary sedimentation of these participants, suggesting that the tablets containing 200 and 400 mg grape seed extracts are safe to consume [45]. Similarly, a subchronic oral toxicity study by Yamakoshi et al. [7] documented that there was no noticeable sign of toxicity in rat fed with grape seed extract as a dietary admixture at 0.02% (low dose group), 0.2% (middle dose group), and 2% (high dose group) (w/w) for 90 days. The authors [7] reported that the NOAEL of grape seed extract used in the subchronic oral toxicity study was 2% in the diet, which was equivalent to 1410 mg/kg body weight in males and 1501 mg/kg body weight/day in females. Therefore, these studies indicate a lack of toxicity and more systematic toxicological studies on grape seed should be conducted.

## 4. Industrial Applications

Lipid peroxidation is one of the major reasons for the deterioration of food products during processing and storage. Therefore, antioxidants have been added to food products to extend their shelf life. Synthetic antioxidants such as tertiary butylhydroquinone (TBHQ), butylated hydroxyanisole (BHA), and butylated hydroxytoluene (BHT) have restricted use in food products because of their carcinogenic and toxic effects [65]. Therefore, the search for natural antioxidants, particularly of plant origin, has greatly increased in recent years.

Grape seeds can also be exploitable for the preservation of food products. For example, grape seed extracts have been used as a food additive in Japan [7]. A study by Jayaprakasha et al. [36] reported that the grape seed extracts showed good reducing power at a 500 μg/L concentration when using potassium ferricyanide reduction methods. In addition, grape seed extracts also showed 65–90% antioxidant activity at 100 ppm.

Grape seed extracts have been evaluated for their antioxidative effect on meat products [66,67]. Ahn et al. [66] reported that the addition of grape seed extracts had an improved oxidative stability and reduced the hexanal concentration by 97% in cooked ground beef after three days of refrigerated storage when compared to the control without added antioxidant. Similarly, Lau and King [67] also reported similar improve oxidative stability in turkey patties. In addition, the authors [67] also reported that when the grape seed extracts were added at 1.0% and 2.0% to turkey patties, the thiobarbituric acid reactive substances (TBARS) values were decreased nearly 10-fold when compared to the control. The grape seed extracts added to turkey patties were also reported to exhibit wine odor and slightly bitter aftertaste [67].

In a study that investigated the effect of four different concentrations of grape seed extracts (i.e., 0.0, 0.4, 0.8, and 1.6 g/kg) on cooked turkey breast meat, Mielnik et al. [68] reported that the addition of grape seed extracts before cooking significantly improved the lipid stability of turkey breast meat during cooking process and chill storage. The authors [68] reported that the efficiency of grape seed extract to prevent oxidation increased when the concentration of antioxidant was from 0.4 g/kg to 1.6 g/kg. Therefore, it is suggested that grape seed extracts can be a very effective antioxidant in inhibiting lipid oxidation of cooked turkey meat during chill-storage. Similarly, Brannan [69] also demonstrated a reduction in the biomarkers of lipid oxidation and sensory scores for key attributes in ground chicken when the grape seed extracts were added during refrigerated storage.

Brannan and Mah [70] documented that 0.1% grape seed extract was effective in inhibiting the formation of primary (e.g., hexanal and lipid hydroperoxides) and secondary oxidation products (e.g., TBARS) in both raw and cooked meat systems during refrigerated and frozen storage. It is suggested that in vivo mechanism of antioxidant activity for grape seed extracts includes the inhibition of nitrositive stress, stimulation of enzymatic production of nitric oxide [71], and oxygen radical scavenging [72].

The antioxidant activity of grape seed extracts can be influenced by heating conditions such as temperature and time. In a study that evaluated the effect of heating conditions on the antioxidant activity of grape seed extracts, Kim et al. [73] reported that heat treatment significantly increased the concentrations of caffeine and gallocatechin gallate in grape seed extracts, suggesting that heating could be used as a method to improve the antioxidant activity of grape seed extracts. Since polyphenols in grape seeds possess antibacterial activity, grape seeds could be incorporated into the foods in order to prevent the growth of foodborne pathogens such as *L. monocytogenes* [21].

Grape seed extracts are also included in the formulations of cosmetic products for anti-aging reasons. This is because grape seed extracts are rich in proanthocyanidins which have strong free radical scavenging properties [74].

## 5. Conclusions and Future Research

Grape seeds have high antioxidant potential. Their potential health benefits include protection against oxidative damage, and anti-diabetic, anti-cholesterol, and anti-platelet properties. The health benefits of grape seed consumption are thought to arise mainly from bioactivities of their polyphenols. Potential health benefits of dietary polyphenols on major chronic non-communicable diseases have been shown in several meta-analyses [75,76,77,78]. Therefore, the screening of individual polyphenol constituents that exhibit health-promoting properties in grape seed requires further investigation. This is because a cause-effect relationship between the intake of grape seed and its health effects can only be established when the composition of grape seeds is properly characterized and standardized. Furthermore, extensive investigation is needed to examine the effects of adding these beneficial polyphenol constituents from grape seeds using advanced technologies into food systems. Further research is needed to evaluate the effectiveness of grape seed extract in the food ecosystem and to establish their role as an antimicrobial agent in food safety.
